# The Effects of Rhythm and Robotic Interventions on the Imitation/Praxis, Interpersonal Synchrony, and Motor Performance of Children with Autism Spectrum Disorder (ASD): A Pilot Randomized Controlled Trial

**DOI:** 10.1155/2015/736516

**Published:** 2015-12-17

**Authors:** Sudha M. Srinivasan, Maninderjit Kaur, Isabel K. Park, Timothy D. Gifford, Kerry L. Marsh, Anjana N. Bhat

**Affiliations:** ^1^Department of Physical Therapy, Biomechanics and Movement Sciences, University of Delaware, Newark, DE 19713, USA; ^2^Physical Therapy Program, Department of Kinesiology, University of Connecticut, Storrs, CT 06269, USA; ^3^Center for Health, Intervention, and Prevention, Department of Psychology, University of Connecticut, Storrs, CT 06269, USA; ^4^Behavioral Neuroscience Program, Department of Psychology, University of Delaware, Newark, DE 19713, USA

## Abstract

We assessed the effects of three interventions, rhythm, robotic, and standard-of-care, on the imitation/praxis, interpersonal synchrony, and overall motor performance of 36 children with Autism Spectrum Disorder (ASD) between 5 and 12 years of age. Children were matched on age, level of functioning, and services received, prior to random assignment to one of the three groups. Training was provided for 8 weeks with 4 sessions provided each week. We assessed generalized changes in motor skills from the pretest to the posttest using a standardized test of motor performance, the Bruininks-Oseretsky Test of Motor Proficiency, 2nd edition (BOT-2). We also assessed training-specific changes in imitation/praxis and interpersonal synchrony during an early and a late session. Consistent with the training activities practiced, the rhythm and robot groups improved on the body coordination composite of the BOT-2, whereas the comparison group improved on the fine manual control composite of the BOT-2. All three groups demonstrated improvements in imitation/praxis. The rhythm and robot groups also showed improved interpersonal synchrony performance from the early to the late session. Overall, socially embedded movement-based contexts are valuable in promoting imitation/praxis, interpersonal synchrony, and motor performance and should be included within the standard-of-care treatment for children with ASD.

## 1. Introduction

Children with Autism Spectrum Disorder (ASD) have persistent impairments in social communication skills including impaired initiation of social interactions, poor sharing of interests with social partners, impairments in verbal and nonverbal communication skills as well as repetitive and restricted interests including repetitive actions on objects, circumscribed interests, and stereotyped speech [1–4]. In addition, between 50 and 100% of individuals with ASD have motor difficulties including incoordination during gross and fine motor activities, poor balance skills, and clumsy gait patterns [5–10]. They also demonstrate impairments in motor imitation, praxis, and interpersonal synchrony [11–15]. Motor impairments in infancy and early childhood have implications for future social, cognitive, and communication development in autism [16]. For example, impairments in basic gross motor skills such as running, jumping, and hopping as well as poor interpersonal synchrony can limit children's play with peers and restrict their opportunities to build social connections and friendships [5, 16]. Similarly, poor manual motor skills such as pointing, requesting, and reaching have implications for nonverbal modes of communication such as use of gestures and engaging in joint attention bids [17]. Poor imitation and praxis skills will limit children's opportunities to learn and refine complex motor skills such as bicycling, playing soccer, and so forth through observation of others' actions [5, 9, 18–20]. Overall, there is substantial evidence supporting the far-reaching cascading effects of motor difficulties on the core impairments in autism. Not surprisingly, motor skills of children with ASD at age 2 were predictive of optimal outcomes at age 4 [21]. Given this unequivocal evidence on the impact of motor skills in ASD, there is a need for intensive research dedicated towards understanding and remediating the motor impairments in children with ASD.

Impairments in gross and fine motor performance as well as interpersonal synchrony are evident from an early age in individuals with ASD [14, 22–26]. In terms of gross motor performance, children have significant impairments in postural control [27–30], gait patterns [6, 31, 32], and bilateral coordination skills [14, 26]. Similarly, children with ASD have impaired fine motor skills involving object control and manual dexterity [33–36], visuomotor integration [9], and handwriting [37–39]. A recent meta-analysis based on 51 studies comparing children with ASD and typically developing (TD) controls demonstrated a large effect size of 1.2 for motor issues in gait, postural control, motor coordination, upper limb control, and motor planning in ASD [10]. Moreover, overall motor performance is associated with the severity of diagnostic symptoms [8, 40], level of adaptive functioning [41], and level of social withdrawal [29] in autism. Children with ASD also have difficulties in interpersonal synchrony [14]. Interpersonal synchrony involves coordinating one's actions with those of social partners and it requires appropriate social attention, imitation, and turn taking skills [13, 26, 42–47]. For example, during a rocking chair task that examined spontaneous interpersonal synchrony between the parent and their child, children with autism showed significantly less in-phase rocking coordination compared to TD children [14]. Overall, given the pervasive nature of motor impairments, it is critical that goals related to motor performance and interpersonal synchrony be brought to the forefront during planning and implementation of interventions for children with ASD.

Difficulties in imitation have been documented in toddlers and young children with ASD [11, 48, 49] and are thought to continue into adulthood [11, 12, 50, 51]. Poor imitation skills may be associated with the core social communication, affective, and cognitive impairments in ASD [52, 53]. Two different types of imitative actions have been described in the literature. Based on object use, there are transitive actions or actions on objects (e.g., hammering a nail) and intransitive actions or actions without objects (e.g., clapping hands). Based on purpose, there are meaningful actions (e.g., actions that convey communicative intent such as waving “bye”) and nonmeaningful actions (e.g., noncommunicative actions such as placing hand on chest) [54, 55]. Young children with ASD had lower imitation scores compared to TD children and children with developmental delays, with greater difficulties in imitation of intransitive compared to transitive actions [49]. Similarly, children had greater impairments in imitation of meaningless versus meaningful actions and imitation impairments were associated with children's overall motor ability levels [56]. Deficits in imitation have also been hypothesized to be part of a more generalized impairment in praxis [57, 58]. Praxis is the ability to plan and execute a series of actions/gestures following imitation, on verbal command, and during tool use [57, 58]. High-functioning children with ASD had impairments in production of gestures on imitation, on verbal command, and during tool use compared to TD children, suggestive of a broader impairment in praxis [58]. Taken together, imitation and praxis impairments are associated with language and play skills as well as levels of symptom severity in children with ASD [40, 49, 59–61]. In spite of considerable evidence on motor impairments, there is little previous research supporting the use of movement-based interventions in ASD. However, recently, there has been growing emphasis on the need for early interventions with a focus on active play and enriched movement experiences to promote multisystem development in ASD [5, 18].

Contemporary interventions that target imitation skills include naturalistic and developmental clinician/caregiver-mediated interventions [62, 63], peer-mediated interventions [64, 65], and video-modeling interventions [66]. Naturalistic and developmental interventions aim to teach imitation within naturalistic, developmentally appropriate social interactions [62, 63]. A 10-week randomized controlled trial using clinician-delivered Reciprocal Imitation Training (RIT) techniques such as contingent imitation, linguistic mapping, modeling, prompting, and contingent natural reinforcement led to improvements in imitation skills in the treatment group but not in the control group that received treatment as usual [67]. In contrast, peer-mediated training involves adults prompting children with ASD to imitate the behaviors of their TD peers; such training led to improvements in imitation of actions on objects as well as generalization of learned skills to novel actions within novel environments [64]. Lastly, motivating approaches such as video modeling, where children with ASD are asked to watch a video clip of the target behavior and then imitate the modeled behavior, have led to better learning of skills such as toy play, brushing, and greeting compared to live modeling techniques [66]. Although several contemporary autism interventions target social imitation, there is currently a lack of evidence on interventions targeting gross/fine motor performance and interpersonal synchrony. In the current study, we explored the effects of two 8-week novel, movement-based rhythm and robotic interventions on the overall motor performance, imitation/praxis, and interpersonal synchrony skills of children with ASD.

Recently, novel intervention approaches such as rhythm and robotic therapies have been used to promote social communication skills in children with ASD [68–70]. Children with autism have a predisposition for musical stimuli, find musical experiences very enjoyable, and have intact musical perception in spite of significant impairments in language skills [68, 71, 72]. Therefore, rhythm-based contexts have been used to enhance verbal and nonverbal communication as well as behavioral skills in ASD [68, 69, 73, 74]. For example, a meta-analysis based on 10 studies and 165 individuals with ASD showed improvements in primary core symptoms of autism including social interaction, verbal communication, initiation of behavior, and social-emotional reciprocity as well as secondary outcomes including social adaptation skills and quality of parent-child relationships [73]. Despite considerable evidence on positive effects of rhythmic interventions on core social communication symptoms, interestingly, few studies have used such contexts to facilitate motor skills in children with ASD [75, 76]. For example, a musical social routine focusing on reciprocal imitation between the child and the experimenter led to improved spontaneous word and action imitation in 3 out of 4 children with ASD, with carry-over effects observed in 2 children [75]. In contrast to the limited literature in autism, there is considerable evidence that rhythmic accompaniment using music can promote gross and fine motor skills in TD children [77–81]. A 10-week music and movement program in preschoolers led to greater improvements in locomotor skills assessed on the standardized, Test of Gross Motor Development (TGMD), compared to a control group that engaged in free play [79]. Overall, music-based contexts seem to be a promising tool to facilitate motor skills in children with ASD [74]. In the current study, we systematically examined the effects of a prolonged rhythm intervention on the motor skills of children with ASD using a randomized controlled trial (RCT) design.

Robotic interactions are highly motivating environments for children with ASD due to the simple, predictable, and nonintimidating nature of robots compared to humans [82, 83]. Children can use robots as a “social crutch” to practice turn taking, language, and joint attention skills; it is hypothesized that they would eventually transfer learned skills to interactions with humans [70, 82, 84]. Robot-child interactions have also been used to encourage imitation skills within structured as well as free play environments [83, 85–90]. For example, following 7 weeks of structured training, low-functioning children paired with a robot mediator demonstrated greater shared attention and imitation of facial expressions of the robot compared to children paired with the human mediator [85]. In spite of these promising results, none of the aforementioned studies systematically assessed changes in imitation or praxis performance following training. Furthermore, studies did not use standardized tests to assess changes in overall motor performance following robotic interactions. Our own previous work suggests objective improvements in imitation/praxis and bilateral coordination following a 4-week robot-adult-child intervention protocol using a 7-inch humanoid robot, Isobot (Tony, Inc.) in 15 TD children and 1 child with autism [91–93]. In the present study, we address limitations in the current literature by extending our work to a larger sample of children with ASD and systematically examining the effects of a prolonged robotic intervention using a humanoid robot, Nao, on training-specific measures of imitation/praxis and interpersonal synchrony assessed within the training context, as well as overall motor performance assessed on a standardized test outside the training context.

Taken together, in spite of the growing recognition of motor impairments in ASD, there is currently little evidence on comprehensive intervention programs that target gross and fine motor performance in autism. Moreover, evidence on novel rhythm and robotic therapies is anecdotal and limited in its application due to small sample sizes, limited training durations, lack of use of standardized assessments, and detailed coding schemes, as well as lack of methodological rigor and experimental controls. The current RCT aimed to address these gaps by comparing the effects of prolonged 8-week rhythm and robotic interventions to those of a standard-of-care comparison intervention on the motor skills of children with ASD. Note that we are currently reporting effects of these interventions on the social communication and behavioral skills through other publications. All three groups engaged in imitation-based activities within group contexts. The movement groups engaged in whole-body gross motor and fine motor imitation games to the beat of music whereas the comparison group engaged in sedentary tabletop fine motor imitation activities. We assessed changes in generalized motor performance from pretest to posttest using a standardized test of motor performance, namely, the Bruininks-Oseretsky Test of Motor Proficiency, 2nd edition (BOT-2) [93]. We also assessed changes in training-specific measures of imitation/praxis and interpersonal synchrony from an early to a late training session. We hypothesized that consistent with the training demands, the movement groups would show improvements in gross motor performance (balance and bilateral coordination subtests), whereas the comparison group would demonstrate improvements in fine motor performance (fine motor precision and fine motor integration subtests) on the BOT-2. Due to the strong focus on imitation-based games in all groups, we hypothesized that all groups would demonstrate improvements in training-specific measures of imitation/praxis. Given the nature of the training activities, we assessed interpersonal synchrony only in the rhythm and robot groups and hypothesized that both groups would demonstrate improvements in synchrony with training.

## 2. Method

### 2.1. Participants

**Figure 1:**
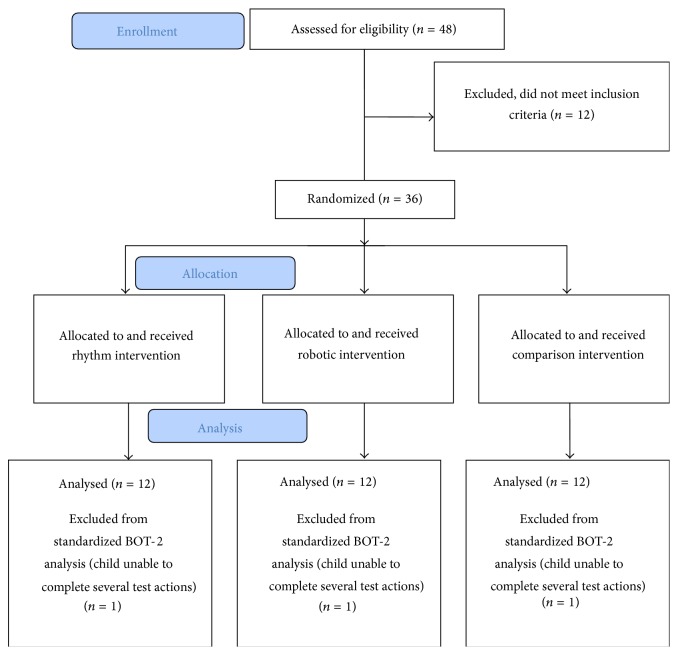
CONSORT flow diagram.

We recruited 36 children (32 M and 4 F, 20 Caucasian, 6 African American, 4 Asian, 3 Hispanic, and 3 of mixed ethnicity) between 5 and 12 years of age (M  (SD) = 7.63  (2.24)) (see Table 1(a) for demographic details) (see Figure 1 for enrollment and allocation details). Children were recruited through fliers posted online and onsite in local schools, services, and self/parent advocacy groups. The Social Communication Questionnaire (SCQ) [94] was used as a screener prior to enrollment. Eligibility was confirmed using the gold standard diagnostic assessment, the Autism Diagnostic Observation Schedule, 2nd edition (ADOS-2) [95], and clinician judgment during a clinical psychology evaluation. Children with significant behavioral impairments or severe receptive language impairments that limited comprehension of 2-step simple instructions were excluded (see Figure 1). All children were enrolled following written parental consent as approved by the Institutional Review Board at the University of Connecticut. All participating families belonged to the upper-middle to upper class in terms of their socioeconomic status (M  (SD) = 49.18  (10.03)) [96] (see Table 1(a)).

Following enrollment, children were matched on age (4-5, 6-7, 8-9, and 10–12 years), level of functioning, and amount of prior services and then randomly assigned to one of three groups, rhythm, robot, or comparison using the random number generator in MS Excel (see Table 1(a)). We assessed autism severity using the comparison scores on the ADOS-2. The comparison score is a continuous metric ranging from 1 to 10 that describes the severity of a child's autism symptoms compared to children with ASD of similar age and language levels [95]. Low comparison scores are indicative of minimal to no evidence of autism symptoms whereas high scores are indicative of severe autism symptoms. All participating children showed moderate to severe symptoms of ASD and there were no group differences in terms of levels of autism severity (*p* values > 0.3, see Table 1(b)). We assessed children's level of adaptive functioning using the Vineland Adaptive Behavior Scales (VABS) parent questionnaire [97] (see Table 1(b)). Overall, 82% of our participants had significant delays (>1 SD below the mean) on the Adaptive Behavior Composite; specifically, 70% children had communication delays, 80% had delays in daily living skills, and 82% had delays on the socialization domain with no group differences (*p* values > 0.5). We used the caregiver checklist of the Movement Assessment Battery for Children, 2nd edition (MABC-2), to assess motor competence of children between 5 and 12 years of age on a variety of fine and gross motor activities [98] (see Table 1(b)). Overall, 75% of children in the study had MABC scores below the 15th percentile on the MABC-2 checklist with no group differences (*p* values > 0.3).

### 2.2. Study Characteristics

Our pilot randomized controlled trial lasted for 10 weeks with the pretest and posttest sessions conducted during the first and last weeks of the study, respectively. The training sessions were provided during the intermediate 8 weeks of the study. Children in all three groups, rhythm, robotic, and comparison, were provided a total of 32 sessions (16 expert and 16 parent sessions). Children were provided 2 expert sessions per week with each session lasting for around 45 minutes. In addition, we provided caregivers with detailed instruction manuals, session supplies, and in-person training to practice similar activities during 2 additional home sessions each week. Out of the 32 sessions, all families completed more than 50% of the sessions (Rhythm: M  (SD) = 73.18  (19.74), Robot: M  (SD) = 76.82  (16.72), Comparison: M  (SD) = 80.21  (15.27), *p* values > 0.05).

### 2.3. Training Protocol

**Figure 2:**
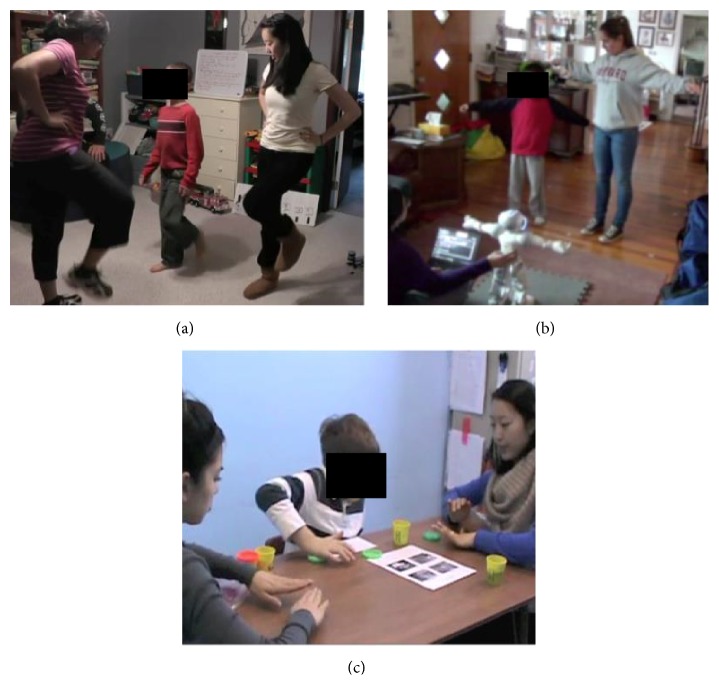
(a) Experimental set-up for a rhythm group training session. (b) Experimental set-up for a robot group training session. (c) Experimental set-up for a comparison group training session.

In all three groups, children engaged in training activities within a triadic context involving the child, an expert trainer, and an adult model (see Figures 2(a), 2(b), and 2(c)). The expert trainer was the instructor and guided the child through the activities of the session. In the robot group, the robot was the instructor and the human trainer controlled the robot using a laptop. The adult model served as a buddy and a visual model for the child and provided hand-on-hand assistance, if needed, during the session. The rhythm and robot groups engaged in socially embedded whole-body movement games (see Figures 2(a) and 2(b)), whereas the comparison group engaged in tabletop activities promoting fine motor, social communication, and academic skills within a group setting (see Figure 2(c)). The comparison group was structured to mimic the types of activities that children with autism typically receive in special education settings. In all three groups, we promoted social communication skills such as eye contact, turn taking, greeting/farewell, responding to questions, commenting, asking for help, and use of gestures. In addition, the rhythm and robot groups promoted gross motor skills including balance, bilateral coordination, imitation, interpersonal synchrony, and manual dexterity during joint action games, whereas the comparison group promoted fine motor skills such as symmetrical and asymmetrical grips and pinches, coloring, drawing, cutting, and gluing. All training sessions were videotaped for further behavioral coding.

In all groups, we used training principles derived from current mainstream autism interventions including Applied Behavioral Analysis (ABA) [99], Teaching and Education of Autistic and Related Communication Handicapped Children (TEACCH) [100], and Picture Exchange Communication System (PECS) [101]. For example, we used strategies such as repetition, graded prompting, ensuring structure and consistency in the environment and the individuals involved in the training, and the use of pictures to facilitate transitions. All trainers involved in the study were pediatric physical therapists or physical therapy/kinesiology graduate students who received intensive training from the last author, a music educator, and ABA experts prior to the training sessions. Similarly, all models involved in the study were undergraduate students experienced in working with children with special needs and they received significant training from the last author prior to participation. To assess treatment fidelity, a naïve coder randomly chose and coded one early (sessions 1–5), mid (sessions 6–11), and late (sessions 12–16) session for each child using a comprehensive checklist developed to assess trainer and model behaviors (see Table 4). The coder evaluated (1) accurate completion of critical components of training activities (maximum score = 74 points), (2) trainer and model behaviors including instructions, prompts, and trainer/model affect (scored on a scale of 1 to 5 with 1 indicating poor quality and 5 indicating highest quality), and (3) child's compliance (scored on a scale of 1 to 5 with 1 indicating poor interest and 5 indicating maximum interest). Overall, across groups, training activities were completed accurately across sessions (Rhythm: 92.16% (8.32), Robot: 90.73% (17.7), Comparison: 91.51% (5.67)), trainers and models demonstrated greater than optimal adherence to the training protocol (Rhythm: 4.68 (0.39), Robot: 4.36 (0.34), Comparison: 4.65 (0.27)), and children showed moderate to high levels of compliance with training (Rhythm: 3.27 (1.14), Robot: 2.67 (0.79), Comparison: 3.95 (0.81)). Next, we describe training activities in each group.

In the rhythm group, children engaged in simple and complex whole-body discrete imitation and interpersonal synchrony-based rhythmic joint action games set to music with the expert trainer and the adult model. Specifically, children engaged in the following movement-based games: action songs that involved finger play, beat keeping routines involving synchronous whole body movements with the adults, improvisational music making that involved synchronous playing of musical instruments, and moving games involving synchronous, locomotor actions such as skipping and jumping (see Table 5).

In the robot group, children engaged with a 23-inch humanoid robot, Nao, and a mobile robot, Rovio, during a variety of dual and multilimb imitation and synchrony-based games. Children practiced the following movement-based activities in each session: warm-up game involving body stretches, action game involving upper and lower body synchrony games, drumming game involving practice of simple and complex drumming patterns, and walking game involving tracing letters and shapes on the floor while following the Rovio robot (see Table 5).

In the comparison group, children engaged in several tabletop activities that promoted academic and fine motor skills. Children engaged in reading developmentally appropriate books, building games that involved making creations using supplies such as Play-Doh, Duplos/Legos, and Zoob (Infinitoy), and art-craft activities involving drawing, cutting, coloring, gluing, and pasting to build theme-based creations. We encouraged fine motor skills including symmetrical and asymmetrical hand movements such as rolling, pressing, pulling apart, pushing together, and different types of grips and pinches, as children engaged in imitation games involving building supplies (see Table 5).

### 2.4. Testing Protocol

We assessed generalized changes in gross and fine motor performance using the standardized test, Bruininks-Oseretsky Test of Motor Proficiency, 2nd edition (BOT-2) [93]. In addition, we assessed training-specific changes in imitation/praxis and interpersonal synchrony by coding custom-developed test activities in the early and late part of the intervention.

#### 2.4.1. Standardized Test of Motor Performance

The BOT-2 is a reliable and valid assessment of gross and fine motor performance for individuals between 4 and 21 years of age [93]. We assessed changes in motor performance using the fine motor precision (FMP), fine motor integration (FMI), bilateral coordination (BC), and balance (BA) subtests of the BOT-2. The FMP subtest consists of 7 activities that assess precise hand and finger control, the FMI subtest includes 8 items that assess the ability to reproduce drawings of geometric shapes, the BC subtest consists of 8 items that assess the ability to sequentially and simultaneously synchronize upper and lower limbs, and the BA subtest consists of 9 items that evaluate postural control skills during standing and walking. We used the standard scores on the body coordination composite and fine manual control composite to assess for training-related changes in motor performance. The body coordination composite is based on the on the BC and BA subtests and the fine manual control composite is based on the FMP and FMI subtests of the BOT-2. A novel tester blinded to the grouping of the child conducted the BOT-2 assessment during the pretest and posttest sessions. The first and the third author coded the entire dataset after establishing inter- and intrarater reliability of greater than 85% using 20% of the dataset. The dependent variable was the standard scores on the body coordination and fine manual control composites of the BOT-2 in the pretest and posttest sessions.

#### 2.4.2. Training-Specific Test of Imitation/Praxis

We developed a set of test actions that were representative of the training activities practiced in each group and administered these activities during an early and late training session. Imitation was assessed during the action song and xylophone games in the rhythm group, during the action and drumming games in the robot group, and during building games using Play-Doh, Duplo/Lego blocks, and Zoob (Infinitoy) pieces in the comparison group (see Table 2 for details). Using video data from the training session, we coded imitation accuracy of children during test actions by assessing errors in spatial and temporal aspects of movement execution relative to actions of the trainer. This coding scheme was developed using the error classification reported in the praxis literature [102] (see Table 3 for details). A score of 0 indicated no error and a score of 1 indicated an error within the specific error category. We also recorded the types of prompts, visual, verbal, or manual hand-on-hand assistance, that children required to complete the actions. The total imitation error score was calculated as the sum of error scores on all individual error categories during the test actions. A single coder coded the entire dataset after establishing inter- and intrarater reliability of over 90% using 20% of the dataset. Our dependent variable was the percent total imitation error during test actions administered in the early and late training sessions in each group.

#### 2.4.3. Training-Specific Test of Interpersonal Synchrony

We assessed changes in interpersonal synchrony between children and their adult partners in the rhythm and robot groups during an early and late training session. Interpersonal synchrony was assessed during the beat keeping, music making, and moving game activities in the rhythm group and during the action, drumming, and walking game activities in the robot group (see Table 2 for details). The comparison group was excluded from this analysis, since they did not engage in whole-body rhythmic actions promoting interpersonal synchrony. In the rhythm and robot groups, we assessed for the percent duration of time that children were in-synchrony (movements synchronized in time and spatially in the same or opposite direction as the adult), out-of-synchrony (movements not synchronized in time and space with the adult), and in assisted-synchrony (manual assistance provided to synchronize movements with the adult) relative to the adult. A single coder coded all the data using Openshapa video coding software after establishing inter- and intrarater reliability of >84% using 20% of the dataset.

### 2.5. Statistical Analysis

We checked our data for assumptions of normality and homogeneity of variances. The data from the standardized BOT-2 test satisfied all assumptions of parametric statistics. We used repeated measures ANCOVA with composite type (body coordination and fine manual control) and test session (pretest and posttest) as within-subjects factors and group as the between-subjects factor. In addition, to control for the effect of autism severity on motor performance, we added the ADOS-2 comparison scores as a covariate in the analysis. Data from our training-specific measure of imitation/praxis and interpersonal synchrony were not normally distributed. Hence, we conducted a square root transformation and used transformed data in our analyses. Moreover, since the tests employed to assess imitation/praxis and synchrony were different in the three groups, we conducted separate analyses for each group. We used dependent *t*-tests to assess changes in imitation/praxis from the early to the late session within each group. For assessing changes in interpersonal synchrony, we conducted separate repeated measures ANOVAs for the rhythm and robot groups with session (early, late) and synchrony type (in-synchrony, out-of-synchrony, assisted-synchrony) as the within-subjects factors. For the ANOVAs, in case of main and interaction effects involving the same factor, we assessed the interaction effects only. In case of violations of the assumption of sphericity, Greenhouse Geisser corrections were applied. Post hoc testing was done using dependent *t*-tests. We report effect sizes using partial eta-squared (*η*_*p*_^2^) and standardized mean difference (SMD) [103] values. Statistical significance was set at *p* ≤ 0.05.

## 3. Results

### 3.1. Standardized Test of Motor Performance

For this analysis, we excluded 3 out of the 36 children since they were low functioning and could not perform a majority of the test actions of the BOT-2, in both the pretest and posttest sessions. The final analysis was therefore based on 11 children per group. The repeated measures ANCOVA indicated significant interaction effect of test session *x* composite type *x* group (*F*(2,29) = 3.44, *p* = 0.05, *η*_*p*_^2^ = 0.19). We report post hoc analysis of the significant interaction as between-group differences and within-group changes.

#### 3.1.1. Between-Group Differences

**Figure 3:**
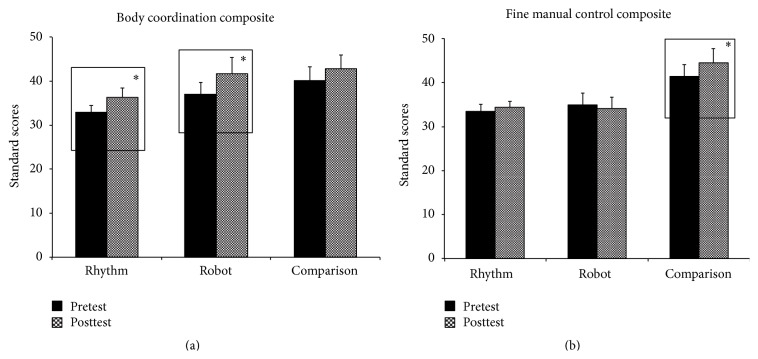
(a) Training-related changes on the body coordination composite of BOT-2. Error bars represent standard errors. (b) Training-related changes on the fine manual control composite of BOT-2. Error bars represent standard errors. ^*∗*^ *p* ≤ 0.05.

At baseline, the comparison group had significantly greater scores on the fine manual control composite than the rhythm group (Rhythm: M  (SD) = 33.5  (5.02), Comparison: M  (SD) = 41.44  (8.72), *p* = 0.02). Based on the pretest session, there were no other baseline differences between groups on the body coordination and fine manual control composite standard scores. In the posttest session, after controlling for baseline levels of autism severity, the comparison group had significantly higher scores on the fine manual control composite compared to the robot and rhythm groups (Rhythm: M  (SD) = 34.38  (4.36), Robot: M  (SD) = 34.13  (8.72), Comparison: M  (SD) = 44.52  (10.82), *p* values < 0.02) (see Figure 3(b)).

#### 3.1.2. Within-Group Changes

In the* rhythm group*, after controlling for baseline levels of autism severity, children improved their scores on the body coordination composite in the posttest compared to the pretest (Pretest: M  (SD) = 32.91  (5.34), Posttest: M  (SD) = 36.36  (6.90), *p* = 0.01, SMD = 0.60) (see Figure 3(a)). Specifically, 9 out of 11 children followed the group trends. Similarly, in the* robot group*, children significantly improved their performance on the body coordination composite in the posttest compared to the pretest, while controlling for the effects of autism severity (Pretest: M  (SD) = 37  (9.15), Posttest: M  (SD) = 41.73  (12.02), *p* = 0.02, SMD = 0.48) (see Figure 3(a)). Specifically, 8 out of 11 children followed the group trends. Children in the rhythm and robot groups did not demonstrate any improvements in fine motor performance (see Figure 3(b)). In the* comparison group*, after controlling for baseline autism severity levels, children significantly increased their scores on the fine manual control composite of the BOT-2 from the pretest to the posttest session (Pretest: M  (SD) = 41.44  (8.72), Posttest: M  (SD) = 44.52  (10.83), *p* = 0.05, SMD = 0.33) (see Figure 2(b)). Individual data show that 9 out of 11 children followed the group trends. This group did not show any improvements on the body coordination composite of the BOT-2 (see Figure 3(a)).

### 3.2. Training-Specific Test of Imitation/Praxis

**Figure 4:**
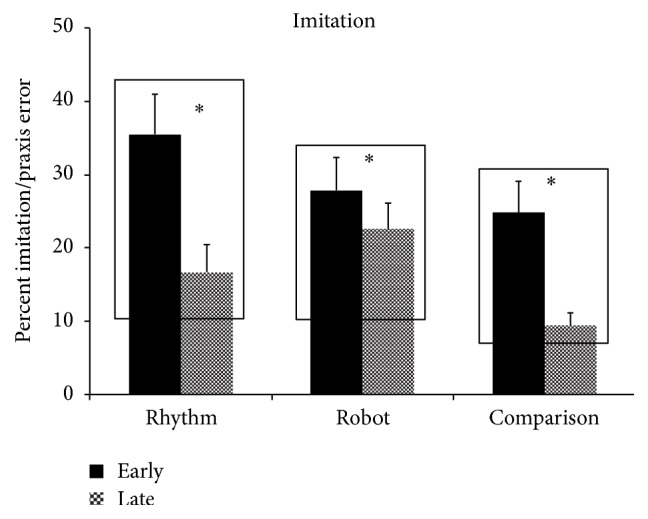
Training-related changes in percent imitation error in the rhythm, robot, and comparison groups. Error bars represent standard errors. ^*∗*^ *p* ≤ 0.05.

In the* rhythm group*, children demonstrated a significant reduction in imitation error scores from the early to the late session (Early: M  (SD) = 35.55  (26.85), Late: M  (SD) = 16.72  (18.21), *t*(23) = 6.16, *p* < 0.001, SMD = −0.65) (see Figure 4). Individual data show that all 12 children in the group demonstrated improvements in imitation performance. In the* robot group*, children significantly improved imitation performance from the early to the late session (Early: M  (SD) = 27.91  (21.66), Late: M  (SD) = 22.60  (17.59), *t*(23) = 2.96, *p* = 0.007, SMD = −0.23) (see Figure 4), with 10 out of the 12 children following the group trends. Along the same lines, the* comparison group* also demonstrated significant improvements in imitation performance from the early to the late session, with 9 out of 12 children following the group trend (Early: M  (SD) = 24.85  (20.69), Late: M  (SD) = 9.38  (8.91), *t*(23) = 4.77, *p* < 0.001, SMD = −0.70) (see Figure 4).

### 3.3. Training-Specific Test of Interpersonal Synchrony

**Figure 5:**
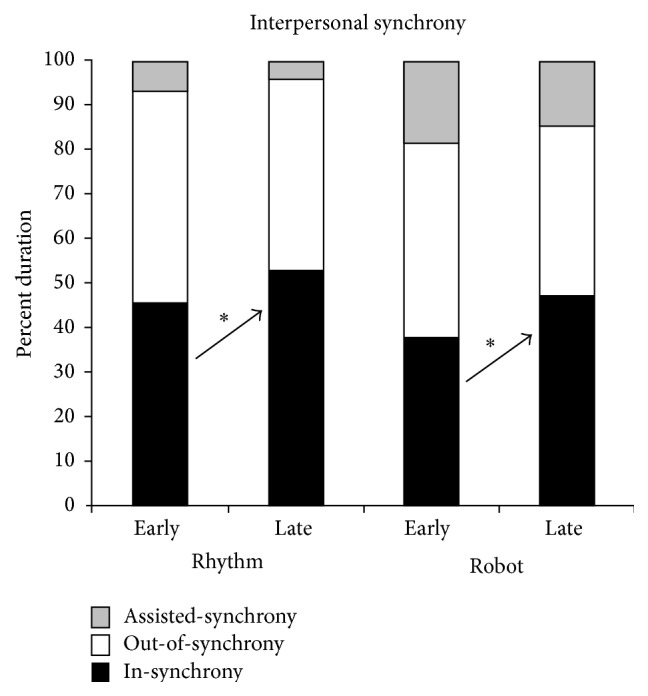
Training-related changes in interpersonal synchrony in the rhythm and robot groups. Error bars represent standard errors. ^*∗*^ *p* < 0.05.

The repeated measures ANOVA in the rhythm group revealed main effects of session (*F*(1,35) = 4.57, *p* = 0.04, *η*_*p*_^2^ = 0.12) and synchrony type (*F*(1.263,44.21) = 51.98, *p* < 0.001, *η*_*p*_^2^ = 0.60) as well as a significant session *x* synchrony type interaction (*F*(2,70) = 3.99, *p* = 0.023, *η*_*p*_^2^ = 0.10). Post hoc analysis of the session *x* synchrony type interaction showed that the rhythm group increased amount of time spent in-synchrony with the adult from the early to the late session (Early: M  (SD) = 45.59  (29.45), Late: M  (SD) = 52.91  (30.57), *p* = 0.02, SMD = 0.23) (see Figure 5). Individual data show that 10 out of the 12 children followed the group trends.

The repeated measures ANOVA in the robot group showed a significant main effect of synchrony type (*F*(1.35,47.22) = 15.66, *p* < 0.001, *η*_*p*_^2^ = 0.31) and a significant session *x* synchrony type interaction (*F*(2,70) = 3.78, *p* = 0.028, *η*_*p*_^2^ = 0.10). Post hoc analysis of the session *x* synchrony type interaction indicated that the robot group increased the amount of time spent in-synchrony with the adult from the early to the late session (Early: M  (SD) = 37.88  (28.55), Late: M  (SD) = 46.99  (30.28), *p* = 0.04, SMD = 0.30), with 10 out of the 12 children following the group trends.

## 4. Discussion

We examined the effects of rhythm and robotic interventions compared to a stationary comparison intervention on the gross and fine motor performance, imitation/praxis, and interpersonal synchrony skills of children with ASD. After controlling for the level of autism severity, the rhythm and robot groups demonstrated improvements in gross motor performance (i.e., body coordination composite) as measured on the BOT-2 from the pretest to the posttest session with no similar improvements on the fine manual control composite (see Figures 3(a) and 3(b)). Children in both groups also reduced their imitation/praxis errors and improved their interpersonal synchrony skills from the early to the late session (see Figures 4 and 5). Consistent with the type of training provided, the comparison group demonstrated an improvement on the fine manual control composite of the BOT-2 from the pretest to the posttest after controlling for level of autism severity, with no significant changes in gross motor performance (see Figures 3(a) and 3(b)). Moreover, children also significantly reduced their imitation errors from the early to the late session (see Figure 4). In the subsequent sections, we discuss possible reasons for our findings and also highlight the implications of our study results.

### 4.1. Changes in Motor Skills within the Standardized and Training-Specific Tests: Rhythm Group

The rhythm group demonstrated an improvement of medium effect size on the body coordination composite of the BOT-2 and the training-specific test of imitation/praxis as well as a small improvement on the training-specific test of interpersonal synchrony. Our findings fit with the limited existing literature on the use of music therapy in children with ASD and the broader music education literature on the effects of music-based experiences on motor skills of TD children [75, 80, 81, 104, 105]. For example, within the context of one-on-one reciprocal musical imitation games involving music-making, singing, and dancing between children with autism and an adult therapist, 3 out of 4 children demonstrated an increase in spontaneous imitation of actions and words of the therapist [75]. In TD children, music has frequently been used as an accompaniment in physical education programs to improve balance and bilateral coordination skills [77, 79, 106, 107]. For example, a developmentally appropriate music and movement program led to greater improvements in jumping and dynamic balance skills of preschool children compared to a control group that engaged in physical education activities that were not based on music [78]. Similarly, following a music- and movement-based program, TD children improved dual and multilimb coordination skills during galloping, leaping, jumping, and skipping actions compared to a control group [107]. Along the same lines, prolonged musical training also led to improvement in accuracy on a motor sequencing task compared to a control group that did not receive musical instruction [80]. In terms of interpersonal synchrony, even 2.5–4.5 year old TD children were able to synchronize more accurately with their adult human partner compared to a machine during a joint drumming task [108]. Moreover, joint music making experiences such as singing or dancing foster prosocial cooperative/synchronous behaviors in 4-year old TD children [109]. Along these lines, the training activities in the rhythm group involved group-based symmetrical and asymmetrical movements of hands and legs such as jumping, galloping, hopping, and so forth to the beat of music. Across training weeks, children may have improved their visuomotor and bilateral coordination as well as balance skills, their ability to plan and execute multistep sequences, the speed and timing of their motor responses, and their ability to synchronize with social partners; this in turn could have led to improvements on standardized and training-specific measures of motor performance.

### 4.2. Changes in Motor Skills within the Standardized and Training-Specific Tests: Robot Group

The robot group also demonstrated small improvements in motor performance on the standardized and training-specific tests following training. Our previous proof-of-concept study on 14 TD children and 1 child with autism suggested improvements in children's ability to coordinate actions with social partners following 4 weeks of imitation-based training using the 7-inch humanoid robot, Isobot (Tony, Inc.) [92]. In the current study, we were able to replicate our previous work and further extend it to a larger sample of children with ASD. Our findings are also similar to the few other studies within the robotics literature that have used robots to facilitate motor skills in children with ASD [85–87, 110–112]. For example, children with ASD were faster at imitating a reach-to-grasp action following observation of a robotic versus a human model performing the task, whereas the reverse trend was observed in TD children [110]. Similarly, repeated short bouts of interactions with a humanoid doll robot, Robota, over 101 days led to a trend for improved imitation of simple actions of the robot in children with ASD [86]. In a different study, during repeated unconstrained interactions with a child-sized humanoid robot, KASPAR, a minimally expressive teenager with autism learned to imitate the actions of the robot as a part of turn taking games played with the therapist and another child [89]. Although the results of the above studies are encouraging, none of the aforementioned studies assessed motor performance using standardized tests or detailed coding schemes to evaluate changes in spatial and temporal aspects of movement execution following intervention. Our findings add to the current body of knowledge by systematically assessing motor performance using training-specific and standardized measures. Over training weeks, children may have improved their perception of the anthropomorphic characteristics of the robot, their balance, motor imitation, and multilimb coordination skills, as well as their social monitoring abilities; this could have contributed to the improvements in imitation/praxis, interpersonal synchrony, and overall gross motor performance seen in this group.

### 4.3. Changes in Motor Skills within the Standardized and Training-Specific Tests: Comparison Group

The comparison group demonstrated an improvement of small effect size on the fine motor subtests of the BOT-2 as well as an improvement of medium effect size on the training-specific test of imitation following the intervention. Our standard-of-care comparison group was designed to mimic the kind of fine motor activities that children with ASD receive during school-based therapies [113]. These services typically address fine motor problems including handwriting skills (illegible writing, inability to stay on the line, letter reversals, etc.), desk skills (using scissors, folding, cutting, rolling Play-Doh, manipulating small objects, etc.), and organization skills (keeping materials in order, completing homework, remembering sequence of tasks, etc.) [114–116]. In line with the types of object manipulation, manual, and desk skills practiced during training sessions, the comparison group demonstrated improvements in fine motor skills. Our findings also fit with existing literature in children with fine motor problems that reports improvements in fine motor and visuomotor skills following prolonged interventions [115, 117]. For example, an 8-month intervention targeting fine motor problems in 44 preschool children led to improvements in standardized tests assessing in-hand manipulation, eye-hand coordination, fine motor skills, visuomotor skills, and functional performance, with the amount of improvement associated with the intensity of training [115]. Similarly, at one-year follow-up following training to address fine motor, visuomotor, and gross motor skills, children with developmental disabilities showed improvements in visuomotor skills as assessed on standardized tests [117]. Overall, it was encouraging to see improvements in fine motor skills both within and outside the training context following a relatively short-term 8-week intervention program.

## 5. Clinical Implications

We assessed the effects of rhythm, robotic, and standard-of-care comparison interventions on the gross and fine motor performance, imitation/praxis, and interpersonal synchrony skills of children with ASD between 5 and 12 years of age. Consistent with the training demands, the movement groups demonstrated improvements in balance, bilateral coordination, gross motor imitation, and interpersonal synchrony skills on standardized and training-specific tests following the 8-week intervention. Similarly, the comparison group demonstrated improvements in fine motor skills on the standardized BOT-2 test and the training-specific test of imitation. Given the association between motor impairments and core symptoms in ASD [8, 40, 118], it would be critical to include both gross and fine motor goals in the treatment plan of children with ASD. Our data show that socially embedded movement-based contexts such as rhythm and robotic therapies that focus on imitation, bilateral coordination, balance, and synchrony skills are enjoyable for children and can be used to promote gross motor skills in this population. In fact, other data from this study show that movement-based contexts can also promote social communication skills such as social monitoring and verbal communication that are considered core impairments in autism. Therefore, in addition to the existing emphasis on remediating fine motor issues in children with ASD within predominantly sedentary contexts, socially embedded creative movement ideas involving rhythm, dance, and active play must also be introduced in the standard-of-care treatments for children with ASD.

We assessed the effects of interventions on training-specific as well as standardized tests of motor performance. The standardized test involved novel actions that were administered by a novel unfamiliar tester outside the training context during the pretest and posttest sessions. In contrast, the training-specific tests involved activities similar to the training activities and were administered by the familiar trainer within the training context during an early and late session. Improvements in the training-specific tests could therefore be attributed to some extent to familiarity effects. In contrast, improvements in the standardized test are indicative of carryover of skills learned during training sessions to novel contexts. It was encouraging to see that there were training-related improvements in both standardized and training-specific tests in all three groups. Clinicians using similar movement-based games for children with ASD should aim to assess training-related changes in motor skills both within and outside the training context and should further evaluate sustenance of treatment effects at follow-up visits.

In the present study, although both rhythm and robot groups demonstrated improvements in motor skills, we observed several limitations in the robotic technology. First, the robot had a limited movement repertoire and could not train children for complex actions such as running, jumping, galloping, hopping, skipping, or even walking at different speeds. Secondly, the robot's movements were slower, less varied, and less precise than those of a human. Lastly, the robot could not train fine motor skills such as cutting, coloring, and drawing. Therefore, although our results look promising, at this point, we recommend that robots be used as adjunct therapies for children with ASD. Future efforts must be directed towards designing training activities that are engaging and functionally meaningful for children with autism, as well as developing contingent, semiautonomous robots that can adapt to the needs of the children.

## 6. Limitations

In spite of the promising results, our study is limited in many ways including a small sample size, limited intervention duration, limited generalization to activities of daily living, and lack of long-term follow-up. Our study was a preliminary study with a relatively small sample size of 36 children with 12 children per group. Further, there was some group variability in our sample due to the nature of the ASD diagnosis. In terms of behavioral coding, the coders of the training-specific measures were not blinded to the grouping of the child. The limited training duration of 8 weeks may have contributed to the small-to-moderate size improvements on the standardized and training-specific measures as well as the lack of significant between-group differences on the standardized BOT-2 test. Overall, in spite of the encouraging nature of our results, we recommend that they be interpreted with caution until future studies can replicate these results using larger sample sizes and extensive training protocols.

## 7. Conclusions

We assessed the effects of novel rhythm and robotic interventions compared to a standard-of-care comparison intervention on the gross and fine motor performance, imitation/praxis, and interpersonal synchrony skills of children with ASD. To the best of our knowledge, this is the first study to systematically assess the effects of rhythm and robotic interventions on motor skills of children using standardized motor tests and custom-developed coding schemes to evaluate imitation/praxis and interpersonal synchrony. Consistent with the training demands of the contexts, the movement groups demonstrated improvements in gross motor performance, whereas the comparison group demonstrated improvements in fine motor performance on the standardized BOT-2 test. All groups demonstrated improvements on the training-specific test of imitation and both the rhythm and robot groups improved their interpersonal synchrony performance following training. Overall, given the perceptuo-motor impairments in ASD, we argue for the inclusion of goals promoting gross and fine motor proficiency within the treatment plan of children with ASD. Our data showed that embodied, whole-body movement-based activities such as rhythm and robotic therapies are valuable contexts to promote motor skills in children with ASD.

## Figures and Tables

**(a):** 

Participant characteristics	Rhythm group M (SD)	Robot group M (SD)	Comparison group M (SD)	*F* or *χ* ^2^ value	*p* value
Age	7.88 (2.56)	7.52 (2.22)	7.36 (2.02)	0.44	0.65
Gender	10 M, 2 F	11 M, 1 F	11 M, 1 F	0.56	0.76
Socioeconomic status	47.33 (10.86)	47.75 (8.75)	52.46 (10.37)	0.97	0.39

**(b):** 

Participant characteristics	Rhythm group M (SD) and range	Robot group M (SD) and range	Comparison Group M (SD) and range	*F* value	*p* value
Adaptive behavior composite on the VABS	71.45 (11.75) 35 to 94	67.91 (15.01) 41 to 88	75.92 (18.43) 39 to 100	0.80	*p* values > 0.5

Percentage of children with scores lower than 1 SD below the mean	81.82	90.91	66.67		

Total motor scores on the MABC-2 checklist	30.92 (16.40) 7 to 62	37.5 (13.06) 3 to 50	30.5 (14.50) 8 to 50	1.45	*p* values > 0.3

Percentage of children below age-based cut-off scores	75	83.33	66.67		

ADOS-2 comparison score	8.5 (1.24) 6 to 10	7.92 (1.78) 5 to 10	8.42 (1.72) 5 to 10	0.46	*p* values > 0.3

*Note.* On the VABS, higher scores indicate better functioning; on the MABC-2 caregiver checklist, higher scores indicate poor motor performance; on the ADOS-2, and higher comparison scores indicate high autism severity.

**Table 2:** Training-specific measures of imitation/praxis and interpersonal synchrony.

Rhythm group		Robot group		Comparison group	
Test of imitation/praxis					

Action song	(i) Finger play (ii) Whole body discrete movements	Action game	Whole body discrete movements	Play-doh game	(i) Roll (ii) Flatten (iii) Pinch Play-Doh
Xylophone game	(i) Unilateral (ii) Bilateral symmetrical (iii) Bilateral alternating (iv) Crossover patterns	Drumming game	(i) Unilateral (ii) Bilateral symmetrical (iii) Bilateral alternating sequences	Duplos/Lego blocks and Zoob pieces	(i) PUSH (ii) Pull blocks/pieces

Test of interpersonal synchrony					

Beat keeping	(i) Rhythmic symmetrical (ii) Alternating arm actions	Action game	Rhythmic asymmetrical arm and leg actions		
Music making	(i) Rhythmic unilateral (ii) Bilateral symmetrical (iii) Bilateral alternating drum sequences	Drumming game	(i) Rhythmic unilateral (ii) Bilateral asymmetrical drum sequences	Not applicable	
Moving game	(i) Jumping (ii) Stomping (iii) March and clap (iv) Skipping	Walking game	Walking synchronously with the model to follow mobile robot		

*Note.* The comparison group did not engage in interpersonal synchrony activities; hence, no training-specific synchrony tests are reported for this group.

**Table 3:** Coding scheme to assess imitation/praxis errors.

Imitation errors	Definitions
Spatial	Incorrect positioning and orientation of joints involved

Body part	Use of incorrect body parts

Movement modulation	Movements are either insufficient or exaggerated in terms of effort and range of motion compared to the trainer

Movement precision	Incorrect sequence of movements within a pattern including omission of steps or addition of extra steps

Pace	Movements slower or faster than the trainer

Symmetry/reciprocity	Two sides of the body are used incorrectly or immaturely to perform test actions

Mirroring	Child failed to mirror actions of the trainer

**Table 4:** A checklist to assess fidelity of the training sessions.

Checklist criteria	Exemplar behaviors assessed
Eye contact	Trainer and model elicit eye contact from child during social interactions

Ready response	Trainer asks child if he/she is ready before each activity

Use of PECS board	Trainer takes child through the activities of the day using the PECS board

Session theme	Trainer explains the theme of the session to the child Example: “Today's theme is turn taking. When I move you watch, and when I stop it is your turn to move”

Activity introduction using PECS	Trainer introduces activity using picture boards Example: “Let's get ready for music making” while pointing at the picture for “music making”

Help with setup	Trainer and model ask child to help with setup for each activity. Example: “Can you pass that blue Lego block to me…”

Presentation of activity	At the beginning of each activity, trainer gives simple instructions for the activity Example: “Now, we will copy the robot”

Activity-specific bids	Appropriate bids to promote motor and social communication skills during each activity were provided Example: For building activity, “Let's roll the Play-Doh into a ball. Roll with me”

Trials	Trainer asks child to repeat each activity twice

Spontaneous exploration	Trainer and model provide children with opportunities for free play and spontaneous exploration Example: “It is free music time. You can play the drums in any way you want”

Social praise	Trainer and model provide verbal and gestural praise to child as required

Help with cleanup	Trainer and model ask child to help with cleanup of supplies after completion of each activity

Activity completion	After each activity, trainer asks child to move down the picture for the activity on the PECS board

General characteristics in the session	The overall session is evaluated for the following characteristics: number of activities completed, environmental arrangement (supplies in close proximity but out of the sight of the child to avoid distractions), and incremental prompts (visual, verbal, gestural, and lastly manual prompts/assistance provided if child is unable to perform the activity )

Trainer and model behaviors	The trainer's and model's behaviors are evaluated for the following criteria on a scale of 1 to 5: (i) appropriateness of instructions, prompts, and reinforcement, (ii) voice and affect modulation, (iii) appropriateness of movements

Child interest	Child's interest and compliance during session assessed on a scale of 1 to 5.

**Table 5:** An exemplar rhythm, robot, and comparison group training session.

Activity	Rhythm group	Robot group	Comparison group
1	Hello song (i) Child asked to sing to trainer and model (ii) Song: hello everybody, yes indeed	Introduction Child asked to greet the trainer, model, and robot	Introduction Child asked to greet the trainer and model

2	Action song (i) Child asked to sing and engage in finger play (ii) Song: open shut them	Warm-up game Child asked to copy whole-body stretching moves of the Nao robot	Book reading Child reads age-appropriate book while taking turns with trainer and model

3	Beat keeping (i) Child asked to copy trainer during rhythmic arm and leg actions (ii) Song: stop, go, go, go	Action game (i) Child asked to engage in upper and lower body synchrony games with Nao robot and model (ii) Theme: start and stop game	Building (i) Child builds creations using Play-Doh, Lego, and so forth (ii) Theme: make a Lego car

4	Music making (i) Child asked to play instruments like drums, xylophones, cymbals, tambourines, and so forth (ii) Song: jingle jingle jingle jive	Drumming game (i) Child asked to practice simple and complex drum patterns with Nao robot and model (ii) Theme: start and stop game	Arts and crafts (i) Child makes creations by drawing, coloring, cutting, and so forth (ii) Theme: make a vegetable basket

5	Moving game (i) Child asked to copy trainer during gross motor actions like skipping, hopping, jumping, and so forth (ii) Song: on the bridge of Newtown	Walking (i) Child asked to follow Rovio robot with the model to trace letters and shapes on the floor (ii) Theme: tracing letter “L”	Cleanup Child asked to clean up all supplies used for the session

6	Farewell song (i) Child asked to sing to trainer and model (ii) Song: it was good to see you	Farewell Child asked to bid goodbye to the trainer, model, and robot	Farewell Child asked to bid goodbye to trainer and model
